# Distinct association between educational attainment and overweight/obesity in unmarried and married women: evidence from a population-based study in Japan

**DOI:** 10.1186/s12889-017-4912-5

**Published:** 2017-11-25

**Authors:** Keiko Murakami, Takayoshi Ohkubo, Hideki Hashimoto

**Affiliations:** 1 Department of Hygiene and Public Health, Teikyo University School of Medicine, 2-11-1 Kaga, Itabashi-ku, Tokyo, 173-8605 Japan; 2 Department of Health and Social Behavior, School of Public Health, The University of Tokyo, 7-3-1 Hongo, Bunkyo-ku, Tokyo, 113-0033 Japan

**Keywords:** Education, Marital status, Japan, Obesity, Overweight, Social influence, Women

## Abstract

**Background:**

Associations between education and obesity have been consistently reported among women in developed countries, but few studies have considered the influence of marital status and husbands’ education. This study aimed to examine differences in the association between education and overweight/obesity by marital status and to determine the contribution of husbands’ education to overweight/obesity among community-dwelling Japanese women.

**Methods:**

A questionnaire survey was conducted from 2010 to 2011 among residents aged 25–50 years in Japanese metropolitan areas. Of 2145 women who agreed to participate and completed the survey, 582 were unmarried and 1563 were married. Overweight/obesity was defined as body mass index ≥25 kg/m^2^. Multiple logistic regression analysis was conducted to determine whether women’s or their husbands’ education was associated with overweight/obesity after adjusting for age, work status, and equivalent income.

**Results:**

The prevalence of overweight/obesity was 11.9% among unmarried women and 10.3% among married women. Women’s own education was significantly associated with overweight/obesity among unmarried women but not among married women. The multivariate-adjusted odds ratio of high school education or lower compared with university education or higher was 3.21 (95% confidence interval: 1.59–6.51) among unmarried women. Among married women, husbands’ education was significantly associated with overweight/obesity: women whose husbands’ educational attainment was high school or lower had significantly higher odds of overweight/obesity than did those whose husbands had a university education or higher (1.67, 95% confidence interval: 1.10–2.55). Among married women whose educational attainment was college or higher, women whose husbands’ educational attainment was high school or lower had a significantly higher risk for overweight/obesity when compared with women whose husbands’ educational attainment was college or higher.

**Conclusions:**

Associations between women’s own education and overweight/obesity varied by marital status, and husbands’ educational level was important for married women’s overweight/obesity. These findings indicate that the social influences bound to educational background affect women’s overweight/obesity.

## Background

Obesity is a serious health burden because of its association with type II diabetes mellitus, cardiovascular diseases, and some types of cancer [1]. The prevalence of obesity is also known to be strongly determined by socioeconomic status among women. In developed countries where obesity has become an epidemic [1], women with low socioeconomic status, or more specifically with low education, are consistently found to have higher risks of obesity [2, 3]. Diverse causal mechanisms underlying the association between education and obesity have been proposed, such as knowledge about the risks of obesity, class-based norms of thinness, and the influence of social networking in groups [3–5].

The association between education and obesity among women is further complicated by findings indicating that this association may differ by marital status. A few studies have demonstrated an inverse association between husbands’ education and adverse health outcomes such as smoking [6], self-rated health [6–8], or mortality [9–14], whereas other studies found no such association [15, 16]. Spousal concordance for obesity has also been well documented [17], suggesting that the social influence of close members of women’s social networks (i.e., husbands among married women) may play a significant role in shaping their behavioral and health norms. Marriage may bring about a drastic change in the sources of social influence on women’s lifestyle choice. Surprisingly, however, it remains unclear to this point whether unmarried and married women exhibit different patterns in terms of educational inequalities in health-related behaviors, including obesity.

In addition to the independent effects of spousal education, the combination of own education and spousal education has recently attracted attention. Few studies have shown that adverse health outcomes were better predicted by the combination of education for both women and their husbands, compared with their own or their husbands’ education alone [6, 18]. Other studies have indicated that an educational discrepancy between spouses may exert a negative influence on health outcomes [9]. These suggest that it is not knowledge of health through education, but rather social influences and norms that result in educational inequalities in health among married women.

In this study, we explored these issues by comparing the associations between own education and overweight/obesity for unmarried and married women, and by testing the independent effects of husbands’ education, as well as the effect of educational discrepancies between spouses.

## Methods

### Data and participants

We used data from the Japanese Study of Stratification, Health, Income, and Neighborhood (J-SHINE). This dataset has been described elsewhere [19–22]. From October 2010 to February 2011, the J-SHINE survey was carried out in four municipalities in and around the greater Tokyo metropolitan area. Of 13,920 community-dwelling adults aged 25–50 years who were probabilistically selected from the residential registry, survey staff members were able to contact 8408 residents. Valid responses were received from 4317 adults, 2313 of whom were women. We analyzed 2145 respondents with no missing values on the variables used in the analysis, excluding income. The secondary use of the data was approved by the J-SHINE Data Management Committee.

### Measures

Marital status was dichotomized into unmarried (single, divorced, or widowed) and married. Participants reported their own educational attainment. Married women also reported their husbands’ educational attainment. These ranged from (a) junior high school, (b) high school, (c) two-year college or special training school, (d) university, and (e) graduate school. Educational attainment was coded into three categories: high school or lower (a, b), college (c), and university or higher (d, e). Women’s own educational attainment, their husbands’ educational attainment, and combinations of these two variables were used as explanatory variables among married women. Combinations of women’s own and husbands’ educational attainment were generated by categorizing both variables into high school or lower (low; a, b) or college or higher (high; c, d, e) and then creating four groups: 1) both spouses with low education, 2) low-educated women with high-educated husbands, 3) high-educated women with low-educated husbands, 4) and both spouses with high education [20].

Body mass index (BMI) was calculated as self-reported body weight (kg) divided by the square of height (m^2^). Overweight and obesity were defined as BMI ≥25 kg/m^2^ and BMI ≥30 kg/m^2^, respectively, adopting the definition of the World Health Organization [23]. We combined the overweight and obesity groups, because the prevalence of BMI ≥30 kg/m^2^ was very low (2.2% among respondents in this study) and the Japan Society for the Study of Obesity has defined obesity as BMI ≥25 kg/m^2^ [24]. Asian populations generally have a higher proportion of body fat than white people of the same age, sex, and BMI [25].

As covariates, we chose age, work status (working, not working), equivalent income, dietary habits, smoking status, and habitual exercise [2]. In the survey, respondents selected their total annual household income from 15 response categories. Using the OECD-modified equivalence scale [26], household income was adjusted for household size. For respondents whose household income was unknown or missing but who responded on individual income, we used individual income as equivalent income. Income values that were missing after this step were imputed using single imputation based on regression analysis including variables for age, marital status, and work status. Dietary habits were measured using 5 questions (“Do you eat breakfast every day,” “Do you try to eat vegetables,” “Do you try to cut down on sugar and salt intake,” “Do you try to purchase organic vegetables and additive-free food,” and “Do you try to eat nutritionally balanced meals?”) rated on a 5-point scale from 1 (*agree*) to 5 (*disagree*). We summed the scores of these 5 responses to determine a total score (range 5–25) and defined poor dietary habits as a score of ≥16 [21]. Smoking status was categorized as current smoker, ex-smoker, or never-smoker. Habitual exercise was measured by how often participants engaged in ≥10 min of physical activity, excluding incidental ones related to work, commuting, or other non-leisure behaviors, over the past year. The responses were every day, 5–6 days a week, 3–4 days a week, 1–2 days a week, once a month, or seldom, and categorized into 3 groups: ≥1 day a week, once a month, or seldom.

### Statistical analysis

Characteristics of unmarried and married respondents were compared using Student’s *t* test for continuous variables and the chi-squared test for categorical variables. Multiple logistic regression analyses were conducted to examine the association between education and overweight/obesity, using women’s education, husbands’ education, and the combination of these two variables as explanatory variables. First, we examined whether marital status modified the association between women’s education and overweight/obesity by including the interaction term in the models. As the significant interaction for women’s own education and marital status was detected (*P* = 0.022), we conducted analyses separately for unmarried and married women.

For unmarried women, we calculated the odds ratio (OR) and 95% confidence interval (CI) for women’s own education adjusted for age (Model 1). We made further adjustments for work status and equivalent income (Model 2), as well as dietary habits, smoking status, and habitual exercise (Model 3). For married women, these analyses were repeated adding husbands’ education as an explanatory variable.

We estimated the OR and 95% CI according to combinations of the women’s education and their husbands’ education using the group of both spouses with high education as reference, and adjusting for age, work status, and equivalent income, as well as dietary habits, smoking status, and habitual exercise.

All analyses were conducted using Stata 12.0 (StataCorp LP, College Station, TX, USA). For all analyses, a two-tailed *P* < 0.05 was considered statistically significant.

## Results

The characteristics of respondents by marital status are shown in Table 1. Married women were older, less educated and less likely to be working, and had higher incomes than unmarried women. The percentages who were overweight or obese were 11.9% and 10.3% among unmarried and married women, respectively.

**Table 1:** Characteristics of unmarried women (*n* = 582) and married women (*n* = 1563)

	Unmarried(*n* = 582)	Married(*n* = 1563)	*P* value^a^
Age, mean (SD)	33.8 (7.1)	38.7 (6.8)	<0.001
Overweight/obesity^b^, n (%)	69 (11.9)	161 (10.3)	0.30
Women’s educational attainment, n (%)			<0.001
≥ University	263 (45.2)	454 (29.0)	
College	220 (37.8)	725 (46.4)	
≤ High school	99 (17.0)	384 (24.6)	
Husbands’ educational attainment, n (%)			
≥ University		927 (59.3)	
College		287 (18.4)	
≤ High school		349 (22.3)	
Women’s and husbands’ educational attainment, n (%)			
≥ College & ≥ College		995 (63.6)	
≥ College & ≤ High school		184 (11.8)	
≤ High school & ≥ College		219 (14.0)	
≤ High school & ≤ High school		165 (10.6)	
Current working, no (%)	525 (90.2)	901 (57.7)	<0.001
Equivalent income^c^, mean (SD)	3131.1 (2097.9)	3494.0 (1974.3)	<0.001
Poor dietary habits, n (%)	134 (23.0)	191 (12.2)	<0.001
Smoking status, n (%)			<0.001
Never-smoker	429 (73.7)	1038 (66.4)	
Ex-smoker	69 (11.9)	325 (20.8)	
Current smoker	84 (14.4)	200 (12.8)	
Habitual exercise, n (%)			0.25
≥ 1 day /week	235 (40.4)	582 (37.2)	
Once /month	101 (17.3)	258 (16.5)	
Seldom	246 (42.3)	723 (46.3)	

^a^Obtained using Student’s *t* test for continuous variables and the chi-squared test for categorical variables, comparing unmarried and married women

^b^Body mass index ≥25.0 kg/m^2^

^c^Thousand Japanese yen (/year)

Table 2 presents the ORs and 95% CIs for overweight/obesity among unmarried women. Unmarried women whose educational attainment was high school or lower had a significantly higher risk for overweight/obesity when compared with unmarried women whose educational attainment was university or higher; the age-adjusted OR (95% CI) was 3.08 (1.57–6.03), and the OR (95% CI) adjusted for age, work status, and equivalent income was 3.21 (1.59–6.51). No significant difference in the risk of overweight/obesity was detected between college education and university or higher. These associations did not materially change after adjustment for health-related behaviors. Among covariates, poor dietary habits were significantly associated with increased risk of overweight/obesity.

**Table 2:** Odds ratios for overweight/obesity according to education among unmarried women (n = 582)

	Overweight/obesity	Model 1	Model 2	Model 3
	%	OR (95% CI)	OR (95% CI)	OR (95% CI)
Women’s educational attainment				
≥ University	7.6	1.00	1.00	1.00
College	10.9	1.15 (0.60–2.20)	1.15 (0.59–2.23)	1.18 (0.60–2.31)
≤ High school	25.3	3.08 (1.57–6.03)	3.21 (1.59–6.51)	3.04 (1.45–6.38)
Work status				
Working	11.1	1.00	1.00	1.00
Not working	19.3	1.92 (0.93–3.96)	2.30 (1.06–5.00)	2.10 (0.95–4.66)
Equivalent income				
4th quartile (highest)	10.5	1.00	1.00	1.00
3rd quartile	14.4	1.79 (0.85–3.75)	1.61 (0.75–3.44)	1.63 (0.75–3.53)
2nd quartile	8.3	0.89 (0.39–2.06)	0.62 (0.26–1.51)	0.59 (0.24–1.45)
1st quartile	13.6	1.39 (0.65–2.97)	0.97 (0.44–2.17)	1.02 (0.45–2.31)
Dietary habits				
Good	9.8	1.00		1.00
Poor	18.7	2.56 (1.47–4.47)		2.41 (1.32–4.39)
Smoking status				
Never-smoker	11.2	1.00		1.00
Ex-smoker	10.1	0.81 (0.35–1.89)		0.62 (0.25–1.52)
Current smoker	16.7	1.39 (0.72–2.69)		0.89 (0.43–1.85)
Habitual exercise				
≥ 1 day/week	12.3	1.00		1.00
Once/month	10.9	0.99 (0.47–2.10)		1.04 (0.48–2.27)
Seldom	11.8	0.96 (0.55–1.68)		0.86 (0.47–1.55)

*OR* odds ratio, *95% CI* 95% confidence interval

Model 1: adjusted for age

Model 2: adjusted for age, work status and equivalent income

Model 3: adjusted for age, work status, equivalent income, dietary habits, smoking status, and habitual exercise

Table 3 presents the ORs and 95% CIs for overweight/obesity among married women. While women’s own education was not significantly associated with overweight/obesity, husbands’ education was significantly associated with overweight/obesity. Married women whose husbands’ educational attainment was high school or lower had a significantly higher risk for overweight/obesity when compared with married women whose husbands’ educational attainment was university or higher; the age-adjusted OR (95% CI) was 1.78 (1.21–2.61), and the OR (95% CI) adjusted for age, work status, equivalent income, and women’s own education was 1.67 (1.10–2.55). Further adjustment for health-related behaviors slightly reduced the association between husbands’ educational attainment and overweight/obesity, but the significance of the association remained. Among covariates, poor dietary habits and current smoking were significantly associated with increased risk of overweight/obesity.

**Table 3:** Odds ratios for overweight/obesity according to education among married women (n = 1563)

	Overweight/obesity	Model 1	Model 2	Model 3
	%	OR (95% CI)	OR (95% CI)	OR (95% CI)
Women’s educational attainment				
≥ University	7.5	1.00	1.00	1.00
College	11.3	1.48 (0.97–2.26)	1.26 (0.81–1.95)	1.19 (0.76–1.85)
≤ High school	11.7	1.49 (0.93–2.40)	1.13 (0.67–1.90)	0.97 (0.57–1.65)
Husbands’ educational attainment				
≥ University	8.6	1.00	1.00	1.00
College	11.2	1.49 (0.96–2.31)	1.40 (0.89–2.22)	1.33 (0.84–2.11)
≤ High school	14.0	1.78 (1.21–2.61)	1.67 (1.10–2.55)	1.56 (1.02–2.40)
Work status				
Working	10.2	1.00	1.00	1.00
Not working	10.4	1.19 (0.85–1.67)	1.21 (0.86–1.70)	1.29 (0.91–1.83)
Equivalent income				
4th quartile (highest)	9.4	1.00	1.00	1.00
3rd quartile	9.8	1.15 (0.71–1.86)	1.02 (0.62–1.67)	1.01 (0.62–1.67)
2nd quartile	10.8	1.41 (0.87–2.30)	1.15 (0.69–1.92)	1.17 (0.70–1.96)
1st quartile	11.2	1.45 (0.90–2.35)	1.20 (0.72–1.98)	1.18 (0.71–1.96)
Dietary habits				
Good	9.5	1.00		1.00
Poor	16.2	2.13 (1.38–3.29)		1.99 (1.26–3.15)
Smoking status				
Never-smoker	9.2	1.00		1.00
Ex-smoker	10.5	1.30 (0.85–1.97)		1.17 (0.76–1.80)
Current smoker	16.0	1.95 (1.26–3.03)		1.63 (1.02–2.60)
Habitual exercise				
≥ 1 day/week	10.1	1.00		1.00
Once/month	12.0	1.35 (0.84–2.15)		1.35 (0.84–2.17)
Seldom	9.8	1.11 (0.77–1.61)		0.97 (0.66–1.42)

*OR* odds ratio, *95% CI* 95% confidence interval

Model 1: adjusted for age

Model 2: adjusted for age, work status, equivalent income, and women’s/husbands’ educational attainment

Model 3: adjusted for age, work status, equivalent income, women’s/husbands’ educational attainment, dietary habits, smoking status, and habitual exercise

**Fig. 1:**
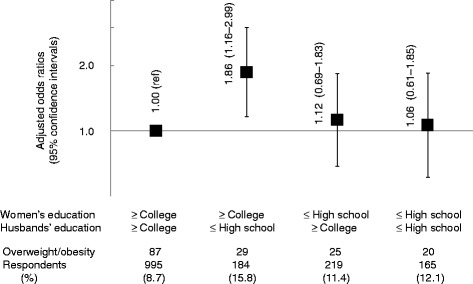
Odds ratios for overweight/obesity according to combination of women’s and husbands’ education among married women (*n* = 1563). Filled squares represent point estimates and horizontal lines denote the 95% confidence interval. Adjusted for age, work status, equivalent income, dietary habits, smoking status, and habitual exercise (Model 3)

Among married women whose educational attainment was college or higher, women whose husbands’ educational attainment was high school or lower had a significantly higher risk for overweight/obesity when compared with women whose husbands’ educational attainment was college or higher (multivariate-adjusted OR = 1.86; 95% CI: 1.16–2.99) (Fig. 1). Low-educated women with high-educated husbands and low-educated women with low-educated husbands did not have significantly higher risk for overweight/obesity compared with high-educated women with high-educated husbands.

## Discussion

The present study explored the associations between education and overweight/obesity among unmarried and married women in Japan. Women’s own education was significantly associated with overweight/obesity among unmarried women, but not among married women. Among married women, husbands’ education was significantly associated with overweight/obesity. In particular, highly educated women who were married to men with low education had a risk of overweight/obesity that was almost double that of highly educated women married to highly educated men.

There are several possible mechanisms through which education may influence health [3–5]. One is that the knowledge and skills attained through education affect cognitive function and therefore susceptibility to health education messages [27]. Education is also considered as a form of cultural capital [3, 28]. Cultural capital in the form of health values and behavioral norms provides the non-material resources needed to promote health behaviors and deal with health problems effectively [28, 29]. Therefore, education, as a form of cultural capital, may have implications for the extent to which an individual is influenced by societal standards of health messages, and those with higher education might prioritize the recognition and pursuit of attributes that are valued in developed societies, such as health and a thin body [3, 28]. In addition, social networks partially account for the association between education and health behaviors [4]. Social networks provide a means by which individual resources such as education can combine with those of others to benefit or disadvantage an individual’s health [30]. Given that people with high socioeconomic status adopt healthy behaviors and associate with others with high socioeconomic status, their networks of social influence promote health within these groups, further widening socioeconomic inequalities in health [4, 5]. Although it remains unclear which aspects of education affect overweight/obesity, our results suggest that the resources obtained from women’s own education affect overweight/obesity only among unmarried women. It is highly unlikely that marriage decreases the effect of the knowledge and skills attained through education.

Unmarried women with college education had a similar risk for overweight/obesity compared with unmarried women with university education or higher. This might be explained by the fact that women are less likely to reach a higher level of tertiary education in Japan, except short-cycle tertiary [31], although the level of educational attainment among Japanese women has been increasing. The share of female graduates is higher at the short-cycle tertiary level (62%) than the average across OECD countries (56%), while at the bachelor’s or equivalent level, 45% of graduates are women in Japan (compared with 58% across OECD countries) [31]. Also in our previous J-SHINE study, the proportions of high school education or lower were similar among men (23.5%) and women (22.5%), but the proportions of university education or higher were significantly higher among men (55.0%) than women (33.6%) [22].

Among married women, husbands’ education was significantly associated with overweight/obesity, even after adjusting for the women’s own education. Spouses are usually genetically unrelated but share a common environment, and spousal concordance for obesity has been demonstrated [17]. People form health values and behavioral norms by comparing their own attitudes with the attitudes of reference groups; they are reinforced when they are shared with the reference groups but altered when they are discrepant [30]. Obesity can follow social networking paths that influence people and cement socioeconomic inequalities in obesity [32]. Women is susceptible to social influence and to the attitudes of those around one [33, 34]. Therefore, education may provide a proxy measure of social group belonging and its norms among women. Marriage is an important dimension of social networks, and this might explain the stronger effects of husbands’ education, compared with women’s own education, on married women’s overweight/obesity risk.

We examined whether health-related behaviors could explain the identified associations between education and overweight/obesity by adjusting for these behaviors in the model. However, adjustment for dietary habits, smoking status, and habitual exercise did not markedly change the associations between education and overweight/obesity, which suggests that these health-related behaviors could not fully explain these associations. However, all data were self-reported, and thus our adjustment could be incomplete. Further studies are needed to explore the mechanisms underlying these associations in more detail.

Educational discrepancy between spouses when highly educated women were married to less educated men resulted in a higher risk of overweight/obesity for the women. There are several explanations for this finding. One is that an unhealthy lifestyle among less educated men may influence their more highly educated wives. As spouses generally share a common environment, exposure to risk factors derived from an unhealthy lifestyle, such as secondhand smoke and dietary habits, may increase the risk of overweight/obesity among highly educated women married to less educated men. Another possible explanation is that this educational discrepancy between spouses, which runs counter to traditionally accepted norms about gender roles, may produce stress and thereby result in negative health consequences. Equivocal results have been reported, especially regarding the effects of a highly educated wife on her husband’s cardiovascular health [35]. One study in Israel found that, for highly educated women, husbands having a lower education almost doubled wives’ risk of mortality from cardiovascular disease; however, husband’s education had no effect among women with lower education [9]. Our results were mostly consistent with this study. Notwithstanding the effects of husbands’ education on health, the health risks associated with educational discrepancy between spouses have rarely been examined. Therefore, our findings further the understanding of the health risks of an educational discrepancy between spouses that runs counter to traditionally accepted norms.

These findings have several implications for health policy. Differences in associations between women’s own education and overweight/obesity by marital status and the importance of husbands’ educational level for women’s overweight/obesity suggest that interventions in public health should pay attention to the social context in which individuals live. Because people belong to households with certain lifestyle and behavior patterns, policies aimed only at individuals may not be successful in influencing people’s behaviors and norms. Our analyses have also suggested that social influences and norms, rather than knowledge and skills attained through education, affect obesity among women. This indicates that prevention programs for obesity should consider women’s susceptibility to social influences in order to reduce educational inequalities in obesity among women. Although obesity is often considered a problem only in Western countries because of the low prevalence of obesity in Asian countries, Asian populations have been shown to have an elevated risk of associated diseases at relatively low BMIs [25]. It would therefore be beneficial to examine educational inequalities in obesity in Japan, where both social structures and body compositions differ from those in Western countries.

Some limitations of this study should be considered. First, the response rate was relatively low. Several studies have found that non-respondents have lower socioeconomic status than do respondents [36, 37]. If such a non-response bias existed in this study, educational inequalities in overweight/obesity would be underestimated. However, the respondents in the present study were fairly comparable with the target population with regard to age, sex, and educational attainment [19]. Second, height and body weight were measured based on self-report, which may cause misclassification. A nationally representative survey in Japan found that self-reported BMI was considerably underestimated among overweight and obese women, mainly because of underreports of weight [38]. If this trend was also present in this study, the observed associations would be underestimated. Third, validated questionnaires would be useful for more precise assessments of dietary habits, although the questions and definition of dietary habits in this study were used also in previous ones [21]. In addition, more detailed questionnaires about physical activities such as sedentary work style would be interesting, as well as habitual exercise. Finally, because this was a cross-sectional study, we could not determine the causal direction of the associations found. It is unlikely that adulthood obesity affects educational attainment. However, adult obesity may have some influence on husbands’ education, because individuals are more likely to marry people who share similar characteristics, such as demographics, attitudes, and behaviors [39]. Unobserved factors associated with marriage selection may confound the association between husbands’ education and obesity.

## Conclusions

The present study found that women’s own education was significantly associated with overweight/obesity among unmarried, but not among married women. Among married women, husbands’ education was significantly associated with overweight/obesity. Highly educated women’s risk of overweight/obesity was almost doubled when they were married to less educated men, compared with highly educated women married to highly educated men. These findings indicate that social influences bound to educational background affect women’s obesity and are important in designing public health intervention to reduce socioeconomic inequalities in obesity and subsequent chronic diseases among women.

## Abbreviations

95% CI: 95% confidence interval
BMI: Body mass index
J-SHINE: Japanese Study of Stratification, Health, Income, and Neighborhood
OR: Odds ratio
